# Kinematic characteristics associated with right-side cross-step blocking performance in elite youth male volleyball players: a cross-sectional study

**DOI:** 10.7717/peerj.21861

**Published:** 2026-11-03

**Authors:** Zhifeng Wei, Huawei Chen, Yiwei Liang, Zipu Wang

**Affiliations:** 1 Department of China Volleyball College, Beijing Sport University, Beijing, China; 2 Capital University of Physical Education and Sports, Beijing, China

**Keywords:** Volleyball, Cross-step blocking, Kinematics, Approach velocity, Jump height, Markerless motion capture, Lower-limb coordination, Adolescent athletes, Lateral movement

## Abstract

**Background:**

Effective blocking is an important contributor to match outcomes in volleyball. However, the phase-specific kinematic characteristics associated with approach velocity (AV) and jump height (JH) during cross-step blocking have not been comprehensively examined. This study aimed to identify the kinematic variables associated with AV and JH during right-side cross-step blocking in elite youth male volleyball players.

**Methods:**

This cross-sectional study included 22 elite youth male volleyball players performing right-side cross-step blocking under standardized conditions, with each participant’s highest-rated eligible trial selected for kinematic analysis based on assessments by two volleyball experts. Kinematic data were recorded using two high-speed video cameras and processed with Fastmove Motion-3D software. Correlation analyses were conducted to identify kinematic variables associated with AV and JH, with *p* values adjusted using the Benjamini–Hochberg false discovery rate (FDR) procedure. Variables that remained significant after FDR correction were subsequently examined using univariable linear regression, followed by parsimonious multivariable linear regression where appropriate.

**Results:**

For AV, five kinematic variables remained significantly associated with AV after FDR correction: third-step right hip linear velocity (R-LVH-TS, *r* = 0.80, FDR *q* < 0.001), second-step left knee angular velocity (L-AVK-SS, *r* = 0.71, FDR *q* = 0.003), second-step left hip linear velocity (L-LVH-SS, *r* = 0.62, FDR *q* = 0.015), second-step foot displacement (SFD, *r* = 0.61, FDR *q* = 0.015), and third-step right knee linear velocity (R-LVK-TS, *ρ* = 0.67, FDR *q* = 0.007). In univariable linear regression models, these variables explained 63.5%, 50.7%, 38.8%, 37.8%, and 24.7% of the variance in AV, respectively. The parsimonious multivariable regression model including third-step right hip linear velocity and second-step left knee angular velocity explained 67.7% of the variance in AV. In this model, only third-step right hip linear velocity remained independently associated with AV. For JH, take-off-phase center-of-mass (CoM) linear velocity was the only variable that remained significantly associated with JH after FDR correction (LVCoM-TO, *ρ* = 0.65, FDR *q* = 0.025), explaining 57.9% of the variance in JH in the univariable regression model.

**Conclusions:**

The findings suggest that AV during right-side cross-step blocking is related to phase-specific lower-limb kinematic coordination during second- and third-step phases, especially right hip linear velocity during the third step. JH is associated with greater CoM linear velocity during take-off. In practice, third-step right hip linear velocity and take-off-phase CoM linear velocity may serve as biomechanical reference points for evaluating approach and jump performance, respectively.

## Introduction

In volleyball, blocking constitutes the first line of defense, with the primary aim of intercepting opponents’ attacks and creating opportunities for counterattacks. Previous studies have reported associations between blocking performance and match outcomes (Mikołajec et al., 2025), particularly during critical points in the fifth set (Giatsis, Drikos & Lola, 2023). Moreover, as the level of competition increases, the frequency and effectiveness of blocking actions become increasingly important to overall match performance (Yu et al., 2018; Echeverría, Ortega & Palao, 2020).

Biomechanical research on volleyball blocking has focused extensively on lower-limb injury mechanisms. Zahradnik et al. (2020) compared anterior cruciate ligament (ACL) risk factors at initial ground contact and at the first and second peaks of the vertical ground reaction force during unanticipated blocking movements performed with different arm positions. Zhao et al. (2024) reported direction- and anticipation-dependent differences in lower-limb kinematics and kinetics during volleyball block jumps in elite male players, with movements in the dominant direction characterized by greater stiffness and reduced cushioning, potentially increasing injury risk. Biomechanical differences in landing strategies across skill levels have also been investigated. Garcia et al. (2022) reported that experienced players exhibited greater ranges of ankle dorsiflexion and knee flexion during blocking landings than novice players. De Bleecker et al. (2025) identified greater knee joint work during volleyball jump-landing tasks, including block jumps, as a risk factor for patellar tendinopathy. Hosseini, Gheitasi & Hosseini (2025) reported fatigue-related changes in lower-limb kinematics during unanticipated blocking that may increase landing injury risk.

Blocking performance has also been investigated in relation to footwork and arm-swing strategies. Approach velocity (AV) and jump height (JH) represent important performance outcomes in blocking (Buekers, 1991; Pawlik & Mroczek, 2023; Vurmaz et al., 2025). Among the different blocking footwork techniques, the cross-step facilitates efficient lateral displacement. Compared with slide-step and stationary blocking techniques, the cross-step is associated with shorter movement times and faster positioning when responding to outside attacks (Buekers, 1991). Cabarkapa et al. (2020) reported greater peak concentric force and a higher rate of force development during the cross-step than during other blocking approaches, while Vurmaz et al. (2025) reported the greatest jump height with the cross-step approach. In addition, differences in JH, take-off velocity, and net penetration have been reported among traditional blocking, chicken-wing blocking, and swing blocking techniques (Neves et al., 2011; Ficklin, Lund & Schipper, 2014). More recently, Yarkin et al. (2026) characterized phase-specific acceleration and timing patterns during cross-step blocking.

Despite these findings, previous research has primarily characterized injury-related mechanics and compared different footwork and arm-swing techniques, leaving the phase-specific kinematic characteristics associated with cross-step blocking performance insufficiently understood. Specifically, it remains unclear which kinematic characteristics across the three-step approach are associated with AV and which characteristics during the planting and take-off phases are associated with JH. This gap is particularly relevant to elite youth athletes, whose movement patterns and technical foundations are still being refined during this developmental period. Identifying these associations may provide biomechanical reference points for phase-specific technique evaluation and the identification of training priorities. Accordingly, this study examined the three approach steps and the planting and take-off phases of right-side cross-step blocking to identify kinematic characteristics associated with AV and JH in elite youth male volleyball players. Higher AV was hypothesized to be associated with greater linear and angular velocities of the left lower limb during the second step and of the right lower limb during the third step. Higher JH was hypothesized to be associated with greater lower-limb angular velocities and greater center-of-mass (CoM) linear velocity during take-off.

## Materials & Methods

### Participants

Twenty-two elite youth male volleyball players participated in this study (mean ± SD: age, 16.0 ± 0.8 years; height, 1.92 ± 0.08 m; body mass, 81.1 ± 13.4 kg). Participants were recruited from the Beijing Youth Men’s Volleyball Team and the volleyball team of Beijing No. 101 High School. At the time of data collection, the Beijing Youth Men’s Volleyball Team had ranked among the top three in a national youth volleyball competition, whereas the Beijing No. 101 High School team had won the Beijing Municipal Games. All participants had at least four years of volleyball training experience and trained six days per week for approximately 2 h per session. All participants were participating fully in regular volleyball training and reported no current musculoskeletal injuries that limited their involvement in training or testing. Before enrollment, the study purpose, procedures, and potential risks were explained to all participants and their parents or legal guardians. Written informed consent was obtained from each participant’s parent or legal guardian. The study protocol was approved by the Ethics Committee of the Capital University of Physical Education and Sports (approval no. 2021A45).

### Experimental task and procedures

Participants performed right-side cross-step blocking trials under standardized volleyball-specific testing conditions. The right-side cross-step blocking task was selected based on match-analysis studies showing that attacks frequently originate from zone 4, potentially requiring blockers to perform rightward lateral displacement (Palao, Santos & Ureña, 2007; Afonso et al., 2010; Afonso & Mesquita, 2011). However, direct evidence from Chinese youth male volleyball competitions remains limited.

Before formal testing, participants completed a standardized 30-min warm-up consisting of jogging, dynamic stretching, and rehearsal of the blocking movement, followed by three familiarization trials. Standing CoM height was then measured once in a quiet standing position and used as the individual baseline for calculating JH. Each participant subsequently completed three preliminary right-side cross-step blocking trials, and the mean AV and JH across these trials were used as individual reference values. After a 3-min rest, each participant completed three formal right-side cross-step blocking trials, with 30 s of rest between trials.

A formal trial met the performance criterion only if both AV and JH reached or exceeded the corresponding individual reference values. Two volleyball experts independently rated the technical quality of each formal trial using a five-point scale (Table S1). Inter-rater reliability between the two experts was assessed using a two-way random-effects, absolute-agreement, single-measure intraclass correlation coefficient [ICC (2,1)]. Inter-rater reliability was ICC = 0.873 (95% CI [0.80–0.92]). Trial-to-trial variability in AV and JH across the three formal trials was assessed using within-participant CVs (SD/mean × 100%), with mean CVs of 4.60% and 1.60%, respectively. To investigate the phase-specific kinematic characteristics within a technically well-executed complete movement, the trial with the highest mean expert rating among those meeting the performance criterion was selected for detailed kinematic analysis.

### Experimental setup and data acquisition

To standardize the starting position, movement direction, and intended blocking location, each participant began in a ready position with both feet inside a rectangular area marked on the volleyball court. The rectangle measured 0.825 m × 0.45 m, with its long axis parallel to the net and the court center line. On the participants’ side of the net, the midpoint of the rectangle’s edge closest to the center line was positioned 0.30 m from the midpoint of the court center line along the perpendicular direction. A blue square target was attached to the top band of the net, 1.50 m medial to the right antenna, corresponding to a lateral distance of 3.0 m to the right of the center of the starting rectangle. From the marked starting area near the middle of the court, participants performed the right-side cross-step blocking trials with maximal effort toward the target, as if blocking an opponent’s attack directed above the target (Ficklin, Lund & Schipper, 2014). Before formal testing, five university professors with expertise in volleyball reviewed the protocol for procedural appropriateness.

Two high-speed video cameras (Z CAM E2; Imaginevision Technology Limited, Shenzhen, China) were used to record each blocking trial. Videos were recorded at a sampling frequency of 60 Hz, with a shutter speed of 1/1,000 s and a resolution of 1920 × 1080 pixels. One camera was positioned 1.0 m outside the right net post, whereas the other was positioned at a posterolateral location relative to the capture area. The cameras were oriented to encompass the entire 6 m × 7 m capture area, with an angle of approximately 90° between their optical axes. Both cameras were positioned at a height of 1.2 m.

### Calibration and data processing

Before formal testing, the three-dimensional capture volume was calibrated using a direct linear transformation (DLT) procedure implemented in Fastmove Motion-3D software. The calibration frame was positioned at the center of the capture volume and was fully visible in both camera views. The frame measured 1.82 m × 1.89 m × 1.62 m and contained 28 calibration points. Ground reference markers were used to define the global coordinate system. The *X*-axis was oriented perpendicular to the court center line in the horizontal plane, the *Y*-axis was oriented parallel to the center line in the horizontal plane, and the *Z*-axis was perpendicular to the court surface and directed vertically upward. The mean reprojection error of the camera calibration was 0.00738 m (range, 0.00185–0.01332 m).

Video data were processed using Fastmove Motion-3D (Fastmove, Dalian, China), an artificial intelligence-based markerless motion capture system. Twenty-one anatomical landmarks, including the head, neck, chin, and bilateral shoulders, elbows, wrists, hands, hips, knees, ankles, heels, and great toes, were automatically detected and reconstructed in three dimensions. The validity of the system was previously evaluated by Liu et al. (2021), who compared automatically reconstructed three-dimensional landmark coordinate-time curves with mean curves obtained through manual digitization by experienced researchers. The coefficients of multiple correlation were generally greater than 0.95, and the absolute errors were less than 0.015 m. Additionally, a recent scoping review reported that computer-vision-based motion analysis achieved accuracy within 0.015 m compared with marker-based systems across six studies of moderate quality (Souaifi et al., 2025).

All reconstructed landmark trajectories were visually inspected, and obvious landmark misidentifications were manually corrected when necessary. The corrected three-dimensional coordinate trajectories were filtered using a Butterworth low-pass filter with a cutoff frequency of 10 Hz, consistent with the 10-Hz cutoff that Dal Pupo et al. (2013) selected using residual analysis for 60-Hz kinematic data collected during rapid continuous vertical jumping. Unless otherwise specified, linear-velocity variables were expressed as the resultant magnitudes of their *X*-, *Y*-, and *Z*-axis components. Software-derived joint-angle variables included three-dimensional spatial vector angles formed by three anatomical landmarks, as well as the corresponding angles projected onto the sagittal, frontal, and transverse planes. Joint angular-velocity variables, including composite angular velocity, were obtained directly from the corresponding software-generated time series.

### Movement phase definition and kinematic variables

The right-side cross-step blocking task comprised two movement components: the cross-step approach and the blocking jump. The cross-step approach consisted of three phases: first step (FS), second step (SS), and third step (TS), whereas the blocking jump consisted of two phases: planting (P) and take-off (TO). The key events defining the boundaries of these phases were manually identified frame by frame. These events are illustrated in Fig. 1 and described in Table 1. Four categories of kinematic variables were analyzed: displacement, temporal, velocity, and joint-angle variables.

**Figure 1:**
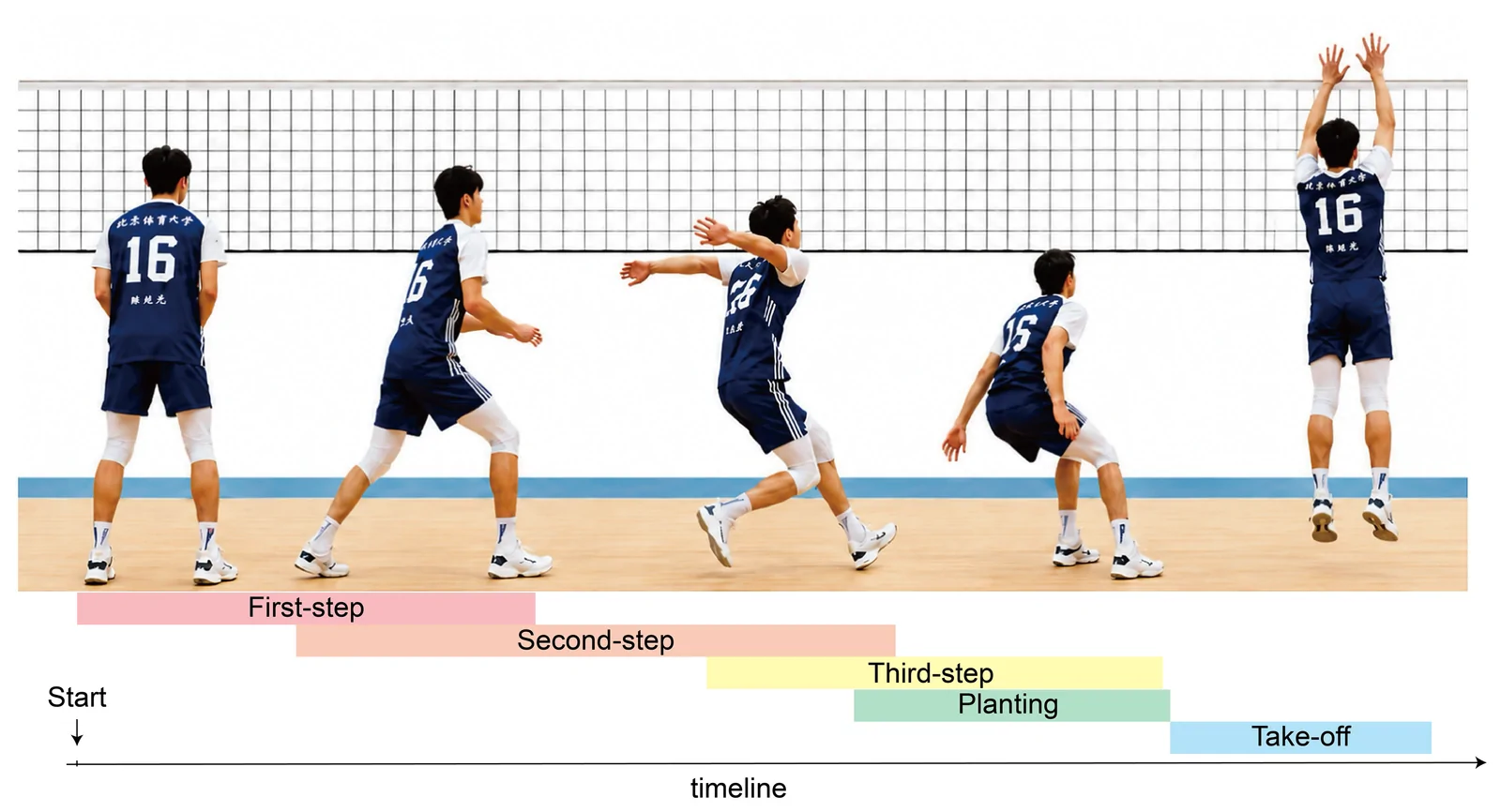
Phase division of the right-side cross-step blocking task. First step: From right-foot toe-off to right-foot contact; Second step: From left-foot toe-off to left-foot contact; Third step: From the second right-foot toe-off to the second right-foot contact; Planting: From left-foot contact to the lowest center-of-mass position; Take-off: From the lowest center-of-mass position to both feet leaving the ground.

**Table 1:** Definitions of movement phases and kinematic variables.

Category	Variable/phase	Definition
Movement phase	First step phase (FS); Second step phase (SS); Third step phase (TS); Planting phase (P); Take-off phase (TO)	FS: from right-foot toe-off to right-foot contact; SS: from left-foot toe-off to left-foot contact; TS: from the second right-foot toe-off to the second right-foot contact; P: from left-foot contact to the lowest CoM position; TO: from the lowest CoM position to the moment when both feet left the ground.
Displacement variables	First foot displacement (FFD); Second foot displacement (SFD); Third foot displacement (TFD); Center of mass displacement (CoMD).	FD was defined as the displacement of the foot during each step of the approach movement. CoMD was defined as the displacement of the center of mass during the jump movement.
Time variables	Time duration of the first step (TDF); Time duration of the second step (TDS); Time duration of the third step (TDT).	TD was defined as the duration of each step of the approach movement.
Velocity variables	Linear velocity of the hip (LVH); Linear velocity of the knee (LVK); Linear velocity of the ankle (LVA); Angular velocity of the hip (AVH); Angular velocity of the knee (AVK); Angular velocity of the ankle (AVA); Linear velocity of the CoM (LVCoM).	Linear- and angular-velocity variables of the hip, knee, and ankle were calculated as the mean values across all frames within each corresponding movement phase. LVCoM was calculated as the mean CoM linear velocity across all frames within each corresponding jump phase.
Joint-angle variables	Hip angle (HA); Knee angle (KA); Ankle angle (AA).	In the planting phase, HA, KA, and AA were measured at the moment when the CoM reached its lowest position, corresponding to the minimum joint angles during this phase. In the take-off phase, HA, KA, and AA were measured at the instant when both feet left the ground, corresponding to the peak joint angles during this phase.

**Notes.**

CoM center of mass FD foot displacement TD time duration

L- and R- denote the left (non-directional) and right (directional) lower limbs, respectively. FS, SS, TS, P, and TO indicate the first step, second step, third step, planting, and take-off phases, respectively. For example, R-LVH-TS refers to right hip linear velocity during the third step phase. The third step phase and planting phase partially overlapped because CoM lowering began before the completion of the third step. First-, second-, and third-step displacement variables were derived from the kinematic data rather than assumed from the fixed target distance.

### Operational definitions of outcomes

Whole-body CoM was automatically calculated within Fastmove Motion-3D using the software’s built-in model based on weighted combinations of reconstructed three-dimensional anatomical landmark coordinates. The equations for the CoM coordinates along the *X*, *Y*, and *Z* axes are provided in Table S4. The resulting CoM time series were used to derive CoM displacement and velocity. JH was defined as the difference between the CoM height at the apex of the blocking jump and the participant’s standing CoM height. AV was defined as the mean CoM velocity along the *Y*-axis from the initiation of the first step to the completion of the third step before take-off. The *Y*-axis represented the primary movement direction for this task.

### Statistical analysis

Kinematic data were analyzed using R version 4.6.1. Descriptive statistics were calculated for all variables. Normally distributed variables were presented as mean ± standard deviation (SD), whereas non-normally distributed variables were presented as median (interquartile range (IQR)). Normality was assessed using the Shapiro–Wilk test.

AV and JH during the right-side cross-step blocking task were treated as continuous performance outcomes. Correlation analyses examined the associations of AV with kinematic variables from the first, second, and third steps and of JH with variables from the planting and take-off phases. Correlations among kinematic variables were also examined separately within each movement phase. Pearson’s correlation coefficient (*r*) was used when both variables were normally distributed; otherwise, Spearman’s rank correlation coefficient (*ρ*) was used. Two-sided 95% confidence intervals for Pearson’s *r* were calculated using Fisher’s *z* transformation, whereas those for Spearman’s *ρ* were estimated using a nonparametric percentile bootstrap method with 2,000 resamples. To account for multiple comparisons, the Benjamini–Hochberg FDR procedure was applied separately to the 33 AV-related correlations and the 22 JH-related correlations. For the phase-specific correlation matrices, FDR correction was applied separately within each phase to all 66 pairwise correlations among the 11 kinematic variables and the corresponding performance outcome. The magnitudes of the absolute correlation coefficients were interpreted according to Cohen’s (1988) criteria as small (0.10 to <0.30), medium (0.30 to <0.50), or large (≥0.50). A sensitivity power analysis indicated that, with a sample size of 22, a two-sided *α* level of 0.05, and 80% statistical power, the smallest detectable correlation before FDR correction was —*r*— = 0.56. This corresponds to a large effect (Cohen, 1988), indicating that the study was primarily powered to detect large associations.

Variables that remained significant after FDR correction were further examined using separate univariable linear regression models to quantify their individual associations with the corresponding performance outcome. When at least two FDR-significant predictors were identified for an outcome, a parsimonious multivariable linear regression model was constructed. To reduce the risk of model overfitting given the sample size, each multivariable model was limited to a maximum of two predictors. Predictors were selected from the FDR-significant variables primarily according to the strength of their univariable associations, while also considering biomechanical relevance and multicollinearity. Multicollinearity was assessed using the variance inflation factor (VIF). Residual and influence diagnostics were examined to assess linearity, the normality and homoscedasticity of residuals, and potentially influential observations. All hypothesis tests were two-sided. Statistical significance was set at *q* < 0.05 for the FDR-corrected correlation analyses and at *p* < 0.05 for the regression analyses.

## Results

AV was 1.53 ± 0.20 m/s (mean ± SD). As shown in Table 2, Table S2, and Fig. S1, five approach-phase kinematic variables remained significantly associated with AV after FDR correction. During the second step, L-LVH-SS, SFD, and L-AVK-SS showed significant positive associations with AV. During the third step, R-LVH-TS and R-LVK-TS were significantly associated with AV. No first-step variables remained significant after FDR correction.

**Table 2:** Descriptive statistics and correlation results for kinematic variables significantly associated with AV after FDR correction (*n* = 22).

Outcome	Predictor	Descriptive value	Coefficient	95% CI	*p*-value	FDR *q*-value
AV	L-LVH-SS	2.26 ± 0.29 m/s	0.62^a^	0.27 to 0.83	0.002	0.015
AV	SFD	1.17 ± 0.20 m	0.61^a^	0.26 to 0.82	0.002	0.015
AV	L-AVK-SS	225.5 ± 64.2°/s	0.71^a^	0.42 to 0.87	<0.001	0.003
AV	R-LVH-TS	2.35 ± 0.28 m/s	0.80^a^	0.57 to 0.91	<0.001	<0.001
AV	R-LVK-TS	3.94 [0.89] m/s	0.67^b^	0.25 to 0.93	<0.001	0.007

**Notes.**

AV approach velocity L-LVH-SS second-step left hip linear velocity SFD second-step displacement L-AVK-SS second-step left knee angular velocity R-LVH-TS third-step right hip linear velocity R-LVK-TS third-step right knee linear velocity

The descriptive value of AV was 1.53 ± 0.20 m/s. Units: L-LVH-SS, R-LVH-TS, and R-LVK-TS are expressed in m/s; SFD is expressed in m; L-AVK-SS is expressed in °/s. Descriptive values are presented as mean ± SD for normally distributed variables and median [IQR] for non-normally distributed variables.

a Pearson’s correlation coefficient.

b Spearman’s rho.

FDR *q*-values were calculated using the Benjamini–Hochberg procedure. Variables with FDR *q* < 0.05 were considered statistically significant.

As shown in Table 3, all five FDR-significant kinematic variables showed significant associations with AV in separate univariable linear regression models and accounted for different proportions of the variance in AV. Specifically, R-LVH-TS, L-AVK-SS, L-LVH-SS, SFD, and R-LVK-TS explained 63.5%, 50.7%, 38.8%, 37.8%, and 24.7% of the variance in AV, respectively. As shown in Table 4, the parsimonious multivariable linear regression model including R-LVH-TS and L-AVK-SS explained 67.7% of the variance in AV. In this model, R-LVH-TS remained significantly associated with AV, whereas the association between L-AVK-SS and AV was no longer statistically significant after adjustment for R-LVH-TS.

**Table 3:** Univariable linear regression models predicting AV from FDR-significant kinematic variables (*n* = 22).

Predictor	B	95% CI for B	*β*	*R* ^2^	Adjusted *R*^2^	*F*	*p*
R-LVH-TS	0.563	0.36 to 0.76	0.797	0.635	0.617	*F*(1, 20) = 34.79	<0.001
L-AVK-SS	0.002	0.001 to 0.003	0.712	0.507	0.482	*F*(1, 20) = 20.57	<0.001
L-LVH-SS	0.424	0.18 to 0.67	0.623	0.388	0.357	*F*(1, 20) = 12.68	0.002
SFD	0.615	0.25 to 0.98	0.615	0.378	0.347	*F*(1, 20) = 12.14	0.002
R-LVK-TS	0.154	0.03 to 0.28	0.497	0.247	0.210	*F*(1, 20) = 6.57	0.019

**Notes.**

R-LVH-TS linear velocity of the right hip during the third step L-AVK-SS angular velocity of the left knee during the second step L-LVH-SS linear velocity of the left hip during the second step SFD second foot displacement R-LVK-TS linear velocity of the right knee during the third step B unstandardized regression coefficient *β* standardized regression coefficient CI confidence interval

Each row represents a separate univariable linear regression model.

**Table 4:** Parsimonious multivariable linear regression model predicting AV (*n* = 22).

Predictor	B	95% CI for B	*β*	*p*-value	VIF
R-LVH-TS	0.415	0.14 to 0.69	0.588	0.005	2.04
L-AVK-SS	0.0009	−0.0003 to 0.002	0.292	0.133	2.04
Model fit: *R*^2^ = 0.677, adjusted *R*^2^ = 0.643, *F*(2, 19) = 19.90, *p* < 0.001.					

**Notes.**

R-LVH-TS linear velocity of the right hip during the third step L-AVK-SS angular velocity of the left knee during the second step B unstandardized regression coefficient *β* standardized regression coefficient CI confidence interval VIF variance inflation factor

JH was 0.73 [0.08] m (median [IQR]). As shown in Table 5, Table S3, and Fig. S1, only LVCoM-TO remained significantly associated with JH after FDR correction, whereas no planting-phase variables remained significant. As shown in Table 6, the univariable linear regression model indicated that LVCoM-TO explained 57.9% of the variance in JH.

**Table 5:** Descriptive statistics and correlation results for the kinematic variable significantly associated with JH after FDR correction (*n* = 22).

Outcome	Predictor	Descriptive value	*ρ*	95% CI	*p*-value	FDR *q*-value
JH	LVCoM-TO	1.85 ± 0.22 m/s	0.65^a^	0.28 to 0.87	0.001	0.025

**Notes.**

JH jump height LVCoM-TO linear velocity of the CoM during the take-off phase

The descriptive value of JH was 0.73 [0.08] m; LVCoM-TO is expressed in m/s, and JH is expressed in m. Descriptive values are presented as mean ± SD for normally distributed variables and median [IQR] for non-normally distributed variables.

a Spearman’s *ρ*.

FDR *q*-values were calculated using the Benjamini–Hochberg procedure. Variables with FDR *q* < 0.05 were considered statistically significant.

**Table 6:** Univariable linear regression model for JH (*n* = 22).

Outcome	Predictor	B	95% CI for B	*β*	*p*-value
JH	LVCoM-TO	0.284	0.17 to 0.40	0.761	<0.001
Model fit: *R*^2^ = 0.579, adjusted *R*^2^ = 0.558, *F*(1, 20) = 27.55, *p* < 0.001.					

**Notes.**

LVCoM-TO linear velocity of the CoM during the take-off phase JH jump height B unstandardized regression coefficient *β* standardized regression coefficient CI confidence interval

The FDR-corrected secondary analyses identified the following significant within-phase correlations. During the second step, L-LVH-SS correlated with L-AVK-SS (*r* = 0.751, *q* = 0.001), L-LVK-SS (*r* = 0.734, *q* = 0.001), L-LVA-SS (*ρ* = 0.587, *q* = 0.014), and TDS (*r* =  − 0.618, *q* = 0.010); L-AVK-SS correlated with L-LVK-SS (*r* = 0.604, *q* = 0.011), L-AVH-SS (*r* = 0.574, *q* = 0.017), and R-AVH-SS (*r* = 0.513, *q* = 0.044); and SFD correlated with TDS (*r* = 0.605, *q* = 0.011). During the third step, R-LVH-TS correlated with R-LVK-TS (*ρ* = 0.631, *q* = 0.010) and L-AVK-TS (*ρ* = 0.538, *q* = 0.045), whereas R-LVK-TS correlated with R-LVA-TS (*ρ* = 0.682, *q* = 0.004), R-AVH-TS (*ρ* = 0.684, *q* = 0.004), R-AVK-TS (*ρ* = 0.612, *q* = 0.014), and TFD (*ρ* = 0.700, *q* = 0.003). During take-off, LVCoM-TO correlated with CoMD-TO (*r* = 0.707, *q* = 0.005).

## Discussion

The principal findings were that third-step right hip linear velocity (R-LVH-TS) was the strongest independent correlate of AV in the parsimonious multivariable model, whereas take-off-phase CoM linear velocity (LVCoM-TO) was the only kinematic variable that remained significantly associated with JH. The hypotheses were partially supported: AV was associated with left lower-limb linear and angular velocities during the second step and right lower-limb linear but not angular velocities during the third step; JH was associated with take-off-phase CoM linear velocity but not lower-limb angular velocities. These findings provide biomechanical insights into right-side cross-step blocking performance in elite youth male volleyball players.

### Approach velocity

During the second step, second-step left hip linear velocity (L-LVH-SS) and second-step left knee angular velocity (L-AVK-SS) were significantly associated with AV and accounted for different proportions of the variance in AV in the univariable regression models. These two variables were also significantly correlated with each other. In addition, L-AVK-SS was significantly associated with second-step left knee linear velocity (L-LVK-SS) and second-step left hip angular velocity (L-AVH-SS), whereas L-LVH-SS was significantly associated with L-LVK-SS and second-step left ankle linear velocity (L-LVA-SS). Collectively, these relationships may reflect coordinated variation among the hip, knee, and ankle of the non-directional lower limb during its forward swing. The significant association between L-AVK-SS and second-step right hip angular velocity (R-AVH-SS) may further reflect bilateral coordination between the forward swing of the non-directional limb and directional-side hip motion during the second step. Finally, L-LVH-SS was negatively associated with second-step time duration (TDS), indicating that greater left hip linear velocity during the second step was related to a shorter step duration. This suggests that left hip velocity during the second step may be associated not only with AV but also with step duration and the temporal characteristics of the second step.

Second-step foot displacement (SFD) was significantly associated with AV in the univariable regression model. Given the limited biomechanical research on cross-step movement, previous studies of the spike approach may provide useful indirect context for comparison. Fuchs et al. (2019) reported a strong positive association between orientation step length and approach velocity during the spike approach. Although the spike approach and cross-step blocking differ in movement direction, with the former mainly involving forward movement and the latter involving lateral displacement, both movements require athletes to approach an optimal take-off position through coordinated step displacement and timing. In the present study, first-, second-, and third-step foot displacements were analyzed during cross-step blocking. Notably, only SFD was significantly associated with AV after FDR correction. This suggests that SFD may represent an important spatial variable associated with lateral movement velocity during cross-step blocking. Further analysis showed that SFD was positively associated with TDS, indicating that greater second-step displacement was accompanied by a longer second-step duration. Despite this longer duration, greater SFD remained associated with higher overall AV. This pattern may be consistent with the second step contributing substantially to lateral displacement and acceleration during the cross-step approach.

During the third step, R-LVH-TS and third-step right knee linear velocity (R-LVK-TS) were significantly associated with AV and were also significantly correlated with each other. In the parsimonious multivariable linear regression model, R-LVH-TS was significantly associated with AV after adjustment for L-AVK-SS. This finding identifies R-LVH-TS as the strongest independent kinematic correlate of AV among the variables included in the model. Further analysis showed that R-LVH-TS was significantly associated with third-step left knee angular velocity (L-AVK-TS). This association may reflect coordinated variation between directional-side hip motion and non-directional knee angular velocity during the third-step closing movement. In addition, R-LVK-TS was significantly associated with third-step right ankle linear velocity (R-LVA-TS), third-step right hip angular velocity (R-AVH-TS), and third-step right knee angular velocity (R-AVK-TS). This suggests that, during the closing movement of the third step, the movement of the directional-side lower limb may involve coordinated actions of the ipsilateral hip, knee, and ankle, reflecting a coupled three-joint movement pattern. Moreover, R-LVK-TS was significantly associated with third-step foot displacement (TFD), indicating that the linear velocity of the directional-side knee may also be related to third-step displacement.

### Jump height

LVCoM-TO appeared to be an important kinematic correlate of JH. Given the limited biomechanical research on blocking-jump kinematics and the partial similarities between blocking and spike-jump movements, studies of the spike jump may provide useful indirect biomechanical context. Sarvestan, Svoboda & Linduška (2020) reported that successful spike performance was characterized by greater vertical CoM velocity and higher JH. FDR-corrected secondary correlation analysis further showed that LVCoM-TO was significantly and positively associated with take-off-phase CoM displacement (CoMD-TO), suggesting that greater CoM displacement during the take-off phase may be accompanied by greater CoM linear velocity during take-off. From a movement-structure perspective, CoM lowering during the planting phase may increase the available vertical displacement for the subsequent upward movement of the CoM during take-off. However, planting-phase CoM displacement (CoMD-P) was not significantly associated with JH in the present study. Therefore, the present data did not provide evidence that the magnitude of CoM lowering during the planting phase was associated with JH. Previous research on spike jumping provides additional indirect biomechanical context for this interpretation. Fuchs et al. (2019) reported that vertical CoM decrease before take-off was associated with JH. Nevertheless, whether greater CoM lowering is beneficial during cross-step blocking may depend on the accompanying movement time and take-off timing, which were not directly evaluated in the present study. Therefore, JH during cross-step blocking may be more closely related to effective upward CoM displacement and greater CoM linear velocity during the take-off phase than to CoM lowering during the planting phase alone. Finally, maturity status may partly contribute to the observed association between LVCoM-TO and JH. Maturity status should therefore be carefully considered when assessing jumping performance in adolescents, particularly in pubertal males (Bchini et al., 2026).

In the present study, none of the hip, knee, or ankle angular-velocity variables remained significantly associated with JH after FDR correction. By contrast, Fuchs et al. (2019) indicated that, during the push-off phase of the spike jump, each 22°/s increase in maximal dominant-knee extension angular velocity was associated with an estimated one-cm increase in jump height. However, because this association was observed during spike jumping, it cannot be directly extrapolated to cross-step blocking. Similarly, none of the hip, knee, or ankle joint-angle variables remained significantly associated with JH after FDR correction. Wagner et al. (2009) found no significant correlations between spike-jump height and hip flexion–extension or ankle dorsiflexion–plantarflexion range-of-motion variables, whereas Sarvestan, Svoboda & Linduška (2020) reported no significant differences in peak knee- or hip-flexion angles between successful and faulty spike trials. These spike-jump findings provide additional indirect biomechanical context but do not establish the absence of associations between joint kinematics and JH during cross-step blocking. Given the limited sample size, further research with larger samples is needed to clarify the contributions of joint angular-velocity and joint-angle variables to blocking-jump performance.

### Strengths and practical applications

A strength of the present study is its phase-specific analysis of the kinematic characteristics associated with right-side cross-step blocking performance in elite youth male volleyball players. Previous studies have mainly focused on comparisons among different blocking techniques, whereas the kinematic characteristics associated with cross-step blocking performance in youth male volleyball players remain insufficiently understood. By treating AV and JH as continuous performance outcomes, this study identified kinematic variables associated with approach and jump performance during right-side cross-step blocking.

From a practical training perspective, the present findings suggest that AV during right-side cross-step blocking should be understood as an integrated movement outcome rather than the result of a single joint action or step-displacement variable. During the second step, coaches may consider the coordination of the non-directional lower limb, with particular attention to left knee angular velocity and second-step displacement, as well as the transition into the subsequent closing step. During the third step, right hip and right knee linear velocities were significantly associated with AV, and the regression results showed that third-step right hip linear velocity remained an independent correlate. This suggests that the coordination of the directional-side lower limb during the third-step phase, especially directional-side hip movement, may be important for AV. The significant association between JH and take-off-phase CoM linear velocity suggests that coaches may pay particular attention to athletes’ ability to generate greater CoM velocity during take-off in cross-step blocking jump training. These practical implications should be interpreted as biomechanical reference points for technique evaluation and the identification of training priorities, rather than as direct evidence of training effects.

### Limitations and future directions

This study has several limitations. First, the inclusion of only elite youth male volleyball players limits the generalizability of the findings to female athletes and adult male players. In addition, unmeasured maturity status may have confounded the observed association between LVCoM-TO and JH. Second, the relatively small sample size may have reduced statistical power, particularly for detecting small-to-moderate associations, and restricted the number of predictors that could be included in the multivariable regression model. In addition, the proportion of variance explained by the regression models is descriptive of the present cohort and may decrease substantially in out-of-sample predictions. Third, detailed phase-specific kinematics were derived only from the highest-rated eligible trial, and their trial-to-trial reliability was not assessed, although AV and JH showed low variability across the three formal trials. Compared with aggregating data from multiple trials, analyzing a single best trial enables characterization of each athlete’s best-trial kinematics but may not fully represent their typical movement pattern. Fourth, the 60-Hz sampling frequency and markerless tracking may have limited the temporal resolution and measurement accuracy of kinematic estimates during rapid phases. Fifth, recruiting participants from only two teams may have introduced team-level clustering effects, as players within each team shared similar training environments, coaching practices, and technical habits. Sixth, the study examined only kinematic variables and did not include kinetic or electromyographic measures, limiting the interpretation of the mechanical and neuromuscular mechanisms underlying cross-step blocking performance. Finally, only right-side cross-step blocking was assessed; therefore, the findings may not generalize to left-side movements. Future studies should include larger and more diverse samples from multiple teams, examine both left- and right-side blocking movements, and incorporate kinetic and electromyographic measurements to provide a more comprehensive understanding of the biomechanical and neuromuscular characteristics associated with cross-step blocking performance.

## Conclusions

Taken together, the findings indicate that AV is significantly associated with second-step left hip linear velocity, left knee angular velocity, and second-step foot displacement, as well as third-step right hip and knee linear velocities. Among these variables, right hip linear velocity during the third-step phase remains the strongest independent correlate of AV in the parsimonious multivariable model. Take-off-phase CoM linear velocity is significantly associated with JH and explains a substantial proportion of its variance.

These results may provide biomechanical reference points for technique evaluation and the identification of training priorities, particularly regarding coordination within the left lower limb and control of step displacement during the second step, coordination between the right hip and knee during the third-step phase, and the generation of greater CoM linear velocity during take-off. In practice, coaches may use greater right hip linear velocity during the third-step phase as a key reference for evaluating approach performance, and greater CoM linear velocity during take-off as a reference for evaluating jump performance. Future studies should include larger samples and incorporate kinetic and electromyographic measurements to determine whether training interventions targeting these kinematic characteristics can improve blocking performance and to clarify the underlying mechanisms.

##  Supplemental Information

10.7717/peerj.21861/supp-1 Figure S1 Phase-specific correlation heatmaps of kinematic variables (A) first step phase correlation heatmap of kinematic variables; (B) second step phase correlation heatmap of kinematic variables; (C) third step phase correlation heatmap of kinematic variables; (D) planting phase correlation heatmap of kinematic variables; (E) take-off phase correlation heatmap of kinematic variables.

10.7717/peerj.21861/supp-2 Table S1 Expert technical rating criteria and scoring scale

10.7717/peerj.21861/supp-3 Table S2 Full correlation analysis between approach-phase kinematic variables and AV

10.7717/peerj.21861/supp-4 Table S3 Full correlation analysis between jump-phase kinematic variables and JH

10.7717/peerj.21861/supp-5 Table S4 Equations for calculating whole-body center-of-mass coordinates

10.7717/peerj.21861/supp-6 Data S1 Raw kinematic data for the jump phase

10.7717/peerj.21861/supp-7 Data S2 Raw kinematic data for the approach phase

10.7717/peerj.21861/supp-8 Supplemental Information 8 README for Raw Data

## Additional Information and Declarations

### Data Availability

The following information was supplied regarding data availability: The raw measurements are available in the Supplemental Files.
