# Supplementary material for: Kinematic characteristics associated with right-side cross-step blocking performance in elite youth male volleyball players: a cross-sectional study
Source: PeerJ. 2026 Nov 3;14:e21861. doi: 10.7717/peerj.21861 (PMC13741976; doi:10.7717/peerj.21861)
Supplement: Supplementary file 1 [file peerj-14-21861-s002.docx]

**Supplementary Table S1:**

**Expert technical rating criteria and scoring scale.**

| Rating item | | Evaluation criteria |
| --- | --- | --- |
| Cross-step movement quality | | Whether the participant completed the prescribed right-side cross-step movement;  whether the starting and final positions were accurate; whether there were no extra adjustment steps, incorrect steps, or missed steps. |
| Jumping movement quality | | Whether the transition from the third step to take-off was continuous;  whether the body was approximately parallel to the net after the third step;  whether there was no net contact or movement interruption. |
| Score | Description | |
| 1 | Clearly failed to meet the technical requirements. | |
| 2 | Poor technical execution with obvious errors. | |
| 3 | Basically met the technical requirements. | |
| 4 | Good technical execution. | |
| 5 | Excellent technical execution. | |

**Note.** Two volleyball experts independently rated each formal trial using the above criteria. Each item was scored on a 5-point scale. Inter-rater reliability was assessed using the intraclass correlation coefficient (ICC).
