# Supplementary material for: Kinematic characteristics associated with right-side cross-step blocking performance in elite youth male volleyball players: a cross-sectional study
Source: PeerJ. 2026 Nov 3;14:e21861. doi: 10.7717/peerj.21861 (PMC13741976; doi:10.7717/peerj.21861)
Supplement: Supplementary file 2 [file peerj-14-21861-s003.docx]

**Supplementary Table S2.**

**Full correlation analysis between approach-phase kinematic variables and AV.**

| Outcome | Predictor | Stage | Descriptive value | Coefficient | 95% CI | p-value | FDR q-value |
| --- | --- | --- | --- | --- | --- | --- | --- |
| AV | R - LVH | TS | 2.35 ± 0.28 | 0.80^a^ | 0.57 to 0.91 | < 0.001 | < 0.001 |
| AV | L - AVK | SS | 225.5 ± 64.2 | 0.71^a^ | 0.42 to 0.87 | < 0.001 | 0.003 |
| AV | R - LVK | TS | 3.94 [0.89] | 0.67^b^ | 0.25 to 0.93 | < 0.001 | 0.007 |
| AV | L - LVH | SS | 2.26 ± 0.29 | 0.62^a^ | 0.27 to 0.83 | 0.002 | 0.015 |
| AV | SFD | SS | 1.17 ± 0.20 | 0.61^a^ | 0.26 to 0.82 | 0.002 | 0.015 |
| AV | L - AVA | FS | 76.5 [62.2] | 0.52^b^ | 0.09 to 0.76 | 0.013 | 0.055 |
| AV | R - AVH | TS | 280.6 [65.5] | 0.52^b^ | 0.09 to 0.75 | 0.014 | 0.055 |
| AV | R - LVA | TS | 5.54 [0.91] | 0.51^b^ | 0.03 to 0.86 | 0.015 | 0.055 |
| AV | L - AVK | TS | 224.9 [42.9] | 0.51^b^ | 0.07 to 0.83 | 0.015 | 0.055 |
| AV | R - LVH | FS | 0.84 [0.31] | 0.50^b^ | 0.00 to 0.84 | 0.018 | 0.055 |
| AV | R - AVA | TS | 144.1 ± 47.2 | 0.50^a^ | 0.10 to 0.76 | 0.019 | 0.055 |
| AV | R - AVK | TS | 268.0 [98.7] | 0.49^b^ | 0.04 to 0.80 | 0.020 | 0.055 |
| AV | R - AVH | SS | 176.8 ± 42.5 | 0.48^a^ | 0.07 to 0.75 | 0.024 | 0.061 |
| AV | L - AVK | FS | 86.1 [41.1] | 0.43^b^ | 0.00 to 0.74 | 0.044 | 0.104 |
| AV | L - AVH | TS | 157.9 ± 40.9 | 0.42^a^ | 0.00 to 0.71 | 0.052 | 0.115 |
| AV | L - AVH | SS | 147.3 ± 41.3 | 0.40^a^ | -0.03 to 0.70 | 0.065 | 0.134 |
| AV | R - LVK | FS | 1.02 ± 0.27 | 0.35^a^ | -0.09 to 0.67 | 0.115 | 0.222 |
| AV | L - LVK | SS | 3.02 ± 0.47 | 0.33^a^ | -0.11 to 0.66 | 0.140 | 0.256 |
| AV | R - AVA | SS | 148.6 ± 33.4 | 0.31^a^ | -0.13 to 0.65 | 0.162 | 0.269 |
| AV | R - AVK | FS | 72.7 ± 30.5 | -0.31^a^ | -0.65 to 0.13 | 0.166 | 0.269 |
| AV | R - LVA | FS | 0.69 [0.23] | 0.30^b^ | -0.19 to 0.73 | 0.176 | 0.269 |
| AV | L - AVH | FS | 64.1 ± 32.3 | 0.30^a^ | -0.14 to 0.64 | 0.179 | 0.269 |
| AV | L - AVA | TS | 114.2 [54.0] | 0.28^b^ | -0.25 to 0.75 | 0.201 | 0.289 |
| AV | TDF | FS | 0.45 ± 0.19 | -0.27^a^ | -0.62 to 0.17 | 0.232 | 0.319 |
| AV | TFD | TS | 0.55 ± 0.13 | 0.25^a^ | -0.20 to 0.60 | 0.272 | 0.358 |
| AV | FFD | FS | 0.38 ± 0.17 | 0.22^a^ | -0.23 to 0.58 | 0.331 | 0.421 |
| AV | R - AVK | SS | 143.9 ± 33.7 | 0.19^a^ | -0.26 to 0.56 | 0.408 | 0.499 |
| AV | L - LVA | SS | 3.52 [1.15] | 0.18^b^ | -0.31 to 0.67 | 0.432 | 0.509 |
| AV | L - AVA | SS | 136.7 ± 36.6 | 0.12^a^ | -0.32 to 0.52 | 0.594 | 0.675 |
| AV | R - AVA | FS | 55.7 [25.9] | -0.10^b^ | -0.56 to 0.40 | 0.647 | 0.711 |
| AV | TDS | SS | 0.59 ± 0.10 | -0.05^a^ | -0.47 to 0.38 | 0.810 | 0.862 |
| AV | TDT | TS | 0.28 ± 0.03 | 0.03^a^ | -0.39 to 0.45 | 0.880 | 0.907 |
| AV | R - AVH | FS | 63.5 ± 26.8 | 0.01^a^ | -0.41 to 0.43 | 0.968 | 0.968 |

Note. AV = approach velocity; FS = first step; SS = second step; TS = third step; L = left; R = right; Descriptive values are presented as mean ± SD for normally distributed variables and median [IQR] for non-normally distributed variables. Units: linear velocities are expressed in m/s, angular velocities in °/s, foot displacement in m, and time duration in s. a = Pearson's correlation coefficient; b = Spearman's rho. FDR q-values were calculated using the Benjamini-Hochberg procedure. Variables with FDR q < 0.05 were considered statistically significant.
