# Supplementary material for: Kinematic characteristics associated with right-side cross-step blocking performance in elite youth male volleyball players: a cross-sectional study
Source: PeerJ. 2026 Nov 3;14:e21861. doi: 10.7717/peerj.21861 (PMC13741976; doi:10.7717/peerj.21861)
Supplement: Supplementary file 3 [file peerj-14-21861-s004.docx]

**Supplementary Table S3:**

**Full correlation analysis between jump-phase kinematic variables and JH.**

| Outcome | Predictor | Stage | Descriptive value | Coefficient | 95% CI | p-value | FDR q-value |
| --- | --- | --- | --- | --- | --- | --- | --- |
| JH | LVCoM | TO | 1.85 ± 0.22 | 0.65 | 0.28 to 0.87 | 0.001 | 0.025 |
| JH | R-LVK | TO | 0.53 ± 0.34 | 0.53 | 0.14 to 0.81 | 0.012 | 0.131 |
| JH | R-LVH | TO | 1.11 ± 0.64 | 0.46 | -0.01 to 0.81 | 0.033 | 0.178 |
| JH | R-AVA | TO | 155.62 ± 56.40 | 0.45 | 0.04 to 0.78 | 0.035 | 0.178 |
| JH | R-AVH | P | 54.17 [50.91] | -0.44 | -0.76 to -0.01 | 0.040 | 0.178 |
| JH | R-LVA | TO | 0.31 ± 0.26 | 0.42 | 0.01 to 0.74 | 0.050 | 0.185 |
| JH | R-AVK | TO | 211.72 [50.29] | 0.41 | 0.01 to 0.75 | 0.059 | 0.186 |
| JH | CoMD | TO | 0.48 ± 0.04 | 0.37 | -0.16 to 0.78 | 0.094 | 0.258 |
| JH | LVCoM | P | 0.54 ± 0.14 | 0.26 | -0.26 to 0.72 | 0.241 | 0.590 |
| JH | R-LVH | P | 1.54 [0.43] | 0.18 | -0.30 to 0.62 | 0.436 | 0.914 |
| JH | R-AVA | P | 238.32 ± 98.44 | 0.16 | -0.28 to 0.58 | 0.487 | 0.914 |
| JH | CoMD | P | 0.13 [0.03] | 0.15 | -0.33 to 0.63 | 0.500 | 0.914 |
| JH | KA | P | 129.55 ± 21.41 | -0.12 | -0.57 to 0.30 | 0.607 | 0.914 |
| JH | HA | P | 124.68 [30.66] | -0.11 | -0.52 to 0.38 | 0.635 | 0.914 |
| JH | HA | TO | 164.70 ± 9.80 | 0.10 | -0.32 to 0.50 | 0.644 | 0.914 |
| JH | KA | TO | 175.33 [2.65] | 0.09 | -0.40 to 0.57 | 0.701 | 0.914 |
| JH | AA | TO | 137.85 ± 8.83 | 0.07 | -0.36 to 0.51 | 0.760 | 0.914 |
| JH | R-AVK | P | 148.65 ± 72.01 | -0.06 | -0.46 to 0.38 | 0.806 | 0.914 |
| JH | AA | P | 97.40 ± 13.24 | -0.05 | -0.51 to 0.35 | 0.816 | 0.914 |
| JH | R-LVA | P | 4.46 [2.33] | -0.05 | -0.47 to 0.37 | 0.843 | 0.914 |
| JH | R-LVK | P | 1.84 [1.09] | -0.04 | -0.52 to 0.45 | 0.873 | 0.914 |
| JH | R-AVH | TO | 52.25 [73.94] | 0.02 | -0.48 to 0.53 | 0.948 | 0.948 |

Note. JH = jump height; P = planting phase; TO = take-off phase. The descriptive value of JH was 0.73 [0.08]. Descriptive values are presented as mean ± SD for normally distributed variables and median [IQR] for non-normally distributed variables. Units: linear velocities and CoM linear velocity are expressed in m/s; angular velocities are expressed in °/s; joint angles are expressed in °; CoM displacement and JH are expressed in m. FDR q-values were calculated using the Benjamini-Hochberg procedure. Variables with FDR q < 0.05 were considered statistically significant.
