# Supplementary material for: Kinematic characteristics associated with right-side cross-step blocking performance in elite youth male volleyball players: a cross-sectional study
Source: PeerJ. 2026 Nov 3;14:e21861. doi: 10.7717/peerj.21861 (PMC13741976; doi:10.7717/peerj.21861)
Supplement: Supplementary file 4 [file peerj-14-21861-s005.docx]

**Z-axis CoM equation**

$$z_{CoM}=\frac{1}{100}\cdot\left[ \begin{aligned} 8.62 \cdot\frac{z_{head}+z_{Neck}}{2} \\ + 44.05 \cdot\frac{2z_{Neck}+z_{RHip}+z_{LHip}}{4} \\ + 14.19 \cdot\frac{z_{RHip}+z_{RKnee}}{2} \\ + 3.67 \cdot\frac{z_{RKnee}+z_{RAnkle}}{2} \\ + 1.48 \cdot\frac{z_{RAnkle}+z_{RBigToe}}{2} \\ + 14.19 \cdot\frac{z_{LHip}+z_{LKnee}}{2} \\ + 3.67 \cdot\frac{z_{LKnee}+z_{LAnkle}}{2} \\ + 1.48 \cdot\frac{z_{LAnkle}+z_{LBigToe}}{2} \\ + 2.43 \cdot\frac{z_{RShoulder}+z_{RElbow}}{2} \\ + 1.25 \cdot\frac{z_{RElbow}+z_{RWrist}}{2} \\ + 0.64 \cdot\frac{z_{RWrist}+z_{Rhand}}{2} \\ + 2.43 \cdot\frac{z_{LShoulder}+z_{LElbow}}{2} \\ + 1.25 \cdot\frac{z_{LElbow}+z_{LWrist}}{2} \\ + 0.64 \cdot\frac{z_{LWrist}+z_{Lhand}}{2} \end{aligned} \right]$$

**Y-axis CoM equation**

$$y_{CoM}=\frac{1}{100}\cdot\left[ \begin{aligned} 8.62 \cdot\frac{y_{head}+y_{Neck}}{2} \\ + 44.05 \cdot\frac{2y_{Neck}+y_{RHip}+y_{LHip}}{4} \\ + 14.19 \cdot\frac{y_{RHip}+y_{RKnee}}{2} \\ + 3.67 \cdot\frac{y_{RKnee}+y_{RAnkle}}{2} \\ + 1.48 \cdot\frac{y_{RAnkle}+y_{RBigToe}}{2} \\ + 14.19 \cdot\frac{y_{LHip}+y_{LKnee}}{2} \\ + 3.67 \cdot\frac{y_{LKnee}+y_{LAnkle}}{2} \\ + 1.48 \cdot\frac{y_{LAnkle}+y_{LBigToe}}{2} \\ + 2.43 \cdot\frac{y_{RShoulder}+y_{RElbow}}{2} \\ + 1.25 \cdot\frac{y_{RElbow}+y_{RWrist}}{2} \\ + 0.64 \cdot\frac{y_{RWrist}+y_{Rhand}}{2} \\ + 2.43 \cdot\frac{y_{LShoulder}+y_{LElbow}}{2} \\ + 1.25 \cdot\frac{y_{LElbow}+y_{LWrist}}{2} \\ + 0.64 \cdot\frac{y_{LWrist}+y_{Lhand}}{2} \end{aligned} \right]$$

**X-axis CoM equation**

$$x_{CoM}=\frac{1}{100}\cdot\left[ \begin{aligned} 8.62 \cdot\frac{x_{head}+x_{Neck}}{2} \\ + 44.05 \cdot\frac{2x_{Neck}+x_{RHip}+x_{LHip}}{4} \\ + 14.19 \cdot\frac{x_{RHip}+x_{RKnee}}{2} \\ + 3.67 \cdot\frac{x_{RKnee}+x_{RAnkle}}{2} \\ + 1.48 \cdot\frac{x_{RAnkle}+x_{RBigToe}}{2} \\ + 14.19 \cdot\frac{x_{LHip}+x_{LKnee}}{2} \\ + 3.67 \cdot\frac{x_{LKnee}+x_{LAnkle}}{2} \\ + 1.48 \cdot\frac{x_{LAnkle}+x_{LBigToe}}{2} \\ + 2.43 \cdot\frac{x_{RShoulder}+x_{RElbow}}{2} \\ + 1.25 \cdot\frac{x_{RElbow}+x_{RWrist}}{2} \\ + 0.64 \cdot\frac{x_{RWrist}+x_{Rhand}}{2} \\ + 2.43 \cdot\frac{x_{LShoulder}+x_{LElbow}}{2} \\ + 1.25 \cdot\frac{x_{LElbow}+x_{LWrist}}{2} \\ + 0.64 \cdot\frac{x_{LWrist}+x_{Lhand}}{2} \end{aligned} \right]$$
